# Role of the bile acid receptor TGR5 (Gpbar-1) in liver damage and regeneration

**DOI:** 10.1186/2047-783X-19-S1-S21

**Published:** 2014-06-19

**Authors:** Verena Keitel, Maria Reich, Annika Sommerfeld, Stefanie Kluge, Ralf Kubitz, Dieter Häussinger

**Affiliations:** 1 Clinic of Gastroenterology, Hepatology and Infectious Diseases, Heinrich Heine University, 40225 Düsseldorf, Germany

## 

Bile acids (BA) are signaling molecules with pleiotropic paracrine and endocrine functions (for recent reviews see [1-4] and references herein). Bile acids are involved in the regulation of bile acid, glucose, lipid and energy homeostasis and can modulate the immune response both in the liver and in the intestine [1,2]. Furthermore, bile acids can promote cell proliferation, cell differentiation and liver regeneration [1-6]. However, especially hydrophobic bile acids may also induce liver damage through induction of programmed cell death (apoptosis) as well as necrosis in hepatocytes [1,2]. Bile acid effects in the liver are cell type and bile acid specific and are facilitated through different bile acid sensing molecules, comprising nuclear hormone receptors, G-protein coupled receptors (GPCRs) as well as integrins and ion channels [1,2].

The farnesoid X receptor (FXR, NR1H4) is a ligand activated transcription factor responsive to different bile acids and highly expressed in hepatocytes. FXR plays an important role in the regulation of bile acid synthesis, detoxification and secretion. Activation of FXR contributes to liver regeneration following partial hepatectomy [5] and alleviates liver injury in different animal models of cholestatic liver disease [1,2].

TGR5 (Gpbar-1, M-Bar) is a GPCR responsive to various unconjugated and conjugated bile acids with taurine-conjugated secondary bile acids, such as taurolithocholic acid (TLC) and taurodeoxycholic acid (TDC), being the most potent TGR5 ligands [1,2]. Expression of TGR5 mRNA is almost ubiquitously detected in human and rodent tissues. In liver, TGR5 is found in sinusoidal endothelial cells (SEC), Kupffer cells (KC), cholangiocytes, gallbladder epithelial cells and gallbladder smooth muscle cells [1-3]. Here, activation of TGR5 by bile acids can alter hepatic microcirculation, modulate the immune response, promote cholangiocyte secretion and induce gallbladder filling [3]. The expression pattern in different non-parenchymal cells of the liver as well as the anti-inflammatory, anti-apoptotic, choleretic and proliferative functions of TGR5 suggest a role for this bile acid receptor in liver injury as well as in liver regeneration [1,3]. Mice with a targeted deletion of TGR5 are more susceptible to develop liver steatosis and hepatic inflammation than wildtype littermates, but are protected from the formation of cholesterol gallstones [3]. Using TGR5 specific agonists different groups could demonstrate that activation of the receptor reduces liver inflammation and steatohepatitis, thereby improving liver function tests [3].

Aim of our studies is to elucidate the role of TGR5 in bile acid induced liver damage and regeneration. TGR5 knockout and wildtype mice were fed ad libitum with a bile acid containing diet for 7 days. Liver injury was assessed by serum biochemistry and liver histology. Cellular proliferation was made visible using immunohistochemistry of liver sections and an antibody against proliferating cell nuclear antigen (PCNA). Ductular proliferation was assessed and quantified by cytokeratin (CK)-19 immunofluorescence staining and confocal laser scanning microscopy. Cholangiocytes were cultivated from isolated ducts from livers of TGR5 wildtype and knockout mice and cell proliferation was measured by BrdU incorporation after stimulation with bile acids or specific TGR5 agonists. The role of TGR5 downstream signalling pathways was analyzed with different kinase inhibitors. The generation of reactive oxygen species (ROS) was measured using a fluorescent dye and shedding of EGF was determined by an ELISA assay. Western blotting was carried out to confirm the phosphorylation of the EGFR and ERK1/2. Bile acid feeding resulted in more elevated liver function tests in TGR5 knockout mice as compared to their wildtype littermates on the same diet. Livers from TGR5 knockout mice fed with bile acids for 4 days showed larger areas of hepatocytes necrosis as livers from wildtype animals on the same diet. In contrast, livers from bile acid-fed wildtype animals displayed increased PCNA-positive hepatocytes and cholangiocytes, as compared to the livers from the respective knockout animals, indicating a rise in cell proliferation following bile acid feeding in wildtype livers. Since TGR5 is highly expressed in cholangiocytes, we focused on bile acid mediated cholangiocyte proliferation. While the amount of CK-19 positive bile ducts was comparable between TGR5 wildtype and knockout mice on chow diet, a significant increase of bile duct proliferation as measured by CK-19 staining was detected in livers from wildtype mice following 7 days on CA diet as compared to livers from TGR5 knockout mice on the same diet.

Incubation of isolated cultivated cholangiocytes from TGR5 wildtype and knockout mice with taurolithocholic acid (TLC 10 and 25 µM) or TGR5 specific agonists led to a significantly increased cholangiocyte proliferation exclusively in wildtype derived cells. Preincubation of wildtype derived cholangiocytes with inhibitors of Src kinases, epidermal growth factor receptor (EGFR) kinase or ERK1/2 kinases significantly reduced TLC and TGR5 agonist mediated cell proliferation as measured by BrDU incorporation. Treatment with inhibitors of ROS formation, such as N-acetylcystein or apocynin also suppressed TLC dependent BrdU incorporation. In contrast, inhibition of adenylate cyclase by SQ22536 or dideoxyadenosine showed no effect on TLC or TGR5 agonist mediated cholangiocyte proliferation. Stimulation of wildtype derived cholangiocytes with TLC and a TGR5 agonist significantly elevated ROS formation. Increased shedding of EGF was also detected after incubation of wildtype cholangiocytes with TLC or the TGR5 agonist. Furthermore, TGR5 activation significantly induced tyrosine phosphorylation of the EGFR at amino acid positions 845 and 1045 and increased ERK1 and ERK2 phosphorylation. Thus, bile acids mediate cholangiocyte proliferation through a TGR5-ROS-Src-EGFR-ERK signaling pathway, which is independent of adenylate cyclase activation.

In summary, TGR5 knockout mice show reduced hepatocyte and cholangiocyte proliferation and more pronounced liver injury in response to bile acid feeding. This is in line with a recent publication, demonstrating that liver regeneration is impaired in TGR5 knockout mice following partial hepatectomy [6].
